# Common genetic predisposition for heart failure and cancer

**DOI:** 10.1007/s00059-020-04953-9

**Published:** 2020-06-15

**Authors:** Tobias J. Pfeffer, Stefan Pietzsch, Denise Hilfiker-Kleiner

**Affiliations:** Department of Cardiology and Angiology, Hannover Medical School, Carl-Neuberg Str. 1, 30625 Hannover, Germany

**Keywords:** Oncocardiology, Cardiovascular disease, Cancer therapy, Genetic susceptibility, Risk factors, Onkokardiologie, Herz-Kreislauf-Erkrankungen, Krebstherapie, Genetische Prädisposition, Risikofaktoren

## Abstract

Cardiovascular diseases and cancer are major causes of mortality in industrialized societies. They share common risk factors (e.g., genetics, lifestyle, age, infection, toxins, and pollution) and might also mutually promote the onset of the respective other disease. Cancer can affect cardiac function directly while antitumor therapies may have acute- and/or late-onset cardiotoxic effects. Recent studies suggest that heart failure might promote tumorigenesis and tumor progression. In both cancer and cardiovascular diseases, genetic predisposition is implicated in the disease onset and development. In this regard, genetic variants classically associated with cardiomyopathies increase the risk for toxic side effects on the cardiovascular system. Genetic variants associated with increased cancer risk are frequent in patients with peripartum cardiomyopathy complicated by cancer, pointing to a common genetic predisposition for both diseases. Common risk factors, cardiotoxic antitumor treatment, genetic variants (associated with cardiomyopathies and/or cancer), and increased cardiac stress lead us to propose the “multi-hit hypothesis” linking cancer and cardiovascular diseases. In the present review, we summarize the current knowledge on potential connecting factors between cancer and cardiovascular diseases with a major focus on the role of genetic predisposition and its implication for individual therapeutic strategies and risk assessment in the novel field of oncocardiology.

## Abstract (de)

Kardiovaskuläre Erkrankungen und Krebserkrankungen gehören zu den häufigsten Todesursachen in der industrialisierten Welt. Beide Krankheitstypen weisen gemeinsame Risikofaktoren (z. B. genetische Prädisposition, Lebensstil, Alter, Infektionen, Toxine und Umweltverschmutzung) auf und können sich gegenseitig begünstigen. Krebserkrankungen beeinträchtigen die Herzfunktion einerseits direkt, und andererseits sind viele onkologische Therapiemethoden mit einem erhöhten Risiko für akute und später auftretende kardiotoxische Nebenwirkungen assoziiert. Umgekehrt gibt es Evidenzen, dass ein geschädigtes Herz die Entstehung und Progression von Tumoren begünstigt. Sowohl für Herz- als auch Krebserkrankungen ist bekannt, dass genetische Prädispositionen die Entstehung und den Krankheitsverlauf beeinflussen. Genetische Varianten in klassischerweise kardiomyopathieassoziierten Genen können zudem die Sensitivität gegenüber kardiotoxischen Chemotherapeutika erhöhen. Patientinnen mit peripartaler Kardiomyopathie haben beispielsweise ein erhöhtes Krebsrisiko, was mit einer gehäuften Prävalenz für krebsprädisponierende Mutationen einhergeht und damit eine gemeinsame genetische Prädisposition für beide Erkrankungen nahelegt. Die gemeinsamen Risikofaktoren, die gegenseitige Beeinflussung durch den genetischen Hintergrund und kardiotoxische Antitumortherapien und erhöhter kardialer Stress sind vermutlich zumindest teilweise für die Koinzidenz von Krebserkrankungen und kardiovaskulären Erkrankungen verantwortlich und führen zur „Multi-Hit-Hypothese“. In diesem Review werden potenzielle Gemeinsamkeiten und Interaktionen zwischen Krebserkrankungen und kardiovaskulären Erkrankungen, mit Fokussierung auf gemeinsame genetische Prädispositionen und die Multi-Hit-Hypothese, zusammengefasst und im Hinblick auf ihre Bedeutung für personalisierte Therapien und Risikobeurteilung diskutiert.

## Background

Cardiovascular diseases and cancer represent the most frequent causes for mortality and morbidity in industrialized countries [1]. Both diseases share common risk factors such as lifestyle and age and many patients are affected by both disease types. Nevertheless, for decades, in clinical routine cardiovascular diseases and cancer have been viewed separately and interdisciplinary treatment concepts were rarely considered. Likewise, intensive basic and clinical research has been performed in both fields and, although cancer and cardiovascular pathologies share many pathways, interdisciplinary cross-talk between researchers in the cancer and cardiovascular field was scarce. However, with the development of highly effective antitumor therapies, the prognosis and long-term survival of cancer patients improved, leading to an increased incidence of cardiovascular problems in these patients [2]. Furthermore, the cardiotoxic side effects of anticancer treatments such as irradiation and anticancer drugs, alone or in combination, became evident and have increasingly moved into the focus of the cardiovascular discipline [3]. Moreover, recent studies discovered interesting genetic interactions between the two disease entities, for example, heart failure was shown to promote transition of pretumor stages and tumor growth [4]. In addition, cancer predisposition syndrome mutations (CPS) are more frequent in patients with peripartum cardiomyopathy (PPCM) compared with age-matched women in the normal population [5].

Based on these connections between cancer and heart failure, interdisciplinary teams with specialized oncocardiology services emerged and oncocardiology became a new clinical and research field [6]. In the present review, we summarize current knowledge on the pathomechanistic connections between cancer and cardiovascular diseases with a specific focus on the role of genetics and the multi-hit hypothesis connecting both disease types.

## Common risk factors

Cancer and cardiovascular diseases display a multifactorial pathogenesis and many aspects of their pathogenesis are shared among the two entities. While some risk factors seem to be cancer specific, i.e., cancer-causing viral infections [7] or specific for cardiomyopathies (high blood pressure, cardiomyopathy-causing gene variants), many other risk factors are common for both disease types (e.g., smoking, metabolic syndrome, irradiation, age, air pollution, and environmental toxins).

However, besides the long list of well-described common risk factors, recent studies showed that cardiovascular diseases and cancer also directly influence each other. In this regard, it is a well-known fact that many anticancer drugs, e.g., anthracyclines (such as epirubicin [8], daunorubicin [9], doxorubicin [10], or idarubicin [11]), antibodies (trastuzumab [12], bevacizumab [13]), or small molecules (such as dasatinib, sunitinib [14], sorafenib [15]) have cardiotoxic side effects and can cause cardiovascular diseases such as heart failure, arrhythmias, atherosclerosis, and thrombosis. These cardiovascular diseases can occur acutely during anticancer treatment or even years after the initial anticancer treatment. Far more than half of all childhood cancer survivors develop treatment-related chronic health issues later in life, of which cardiovascular complications make up a substantial fraction [16].

Another aspect in this context is the potential effect of immunosenescence, a term describing the gradual deterioration of the immune system during aging. Increased levels of inflammatory cytokines, released by senescent cells in the bone marrow niche, contribute to hematopoietic stem cell aging, which can cause an increased susceptibility to infections, cancer, and cardiovascular diseases [17]. A high incidence of age-related clonal hematopoiesis of indeterminate potential (CHIP) mutations might be a result of a senescent hematopoietic system, favoring, e.g., ten-eleven translocation 2 (TET2; [17]). Furthermore, experimental studies revealed that tumors can directly impact on the heart, e.g., by modifying cardiac metabolism resulting in impairment of cardiac function. Reduction of systemic insulin levels in melanoma tumor-bearing mice due to consumption of large amounts of glucose by the tumor tissue interfered with cardiac glucose uptake and was associated with cardiac atrophy and dysfunction [18]. Another study proposed that oncometabolite d‑2-hydroxyglutarate, released by leukemic cells, induced contractile dysfunction and histone modifications [19].

Conversely, experimental data indicate that cardiovascular diseases can also promote the development of cancer diseases, as shown by Meijers et al. that heart failure stimulates the transition of pre-tumor stages and tumor growth in a mouse model, which is caused by cardiac excreted factors [4]. Epidemiological studies further show that prevalent heart failure increases the risk of developing cancer [20–23].

## Genetic background

Besides these well-described risk factors, genetic background plays an important role in the pathogenesis of both entities. Several studies demonstrated that genetics might not only contribute to the pathogenesis of each disease type, but also connect cardiovascular diseases and cancer.

### Somatic mutations

Somatic mutations such as the aforementioned CHIP mutations, which physiologically accumulate during the aging process and are known to increase the risk of hematological neoplasia, became the focus of several working groups in the field of oncocardiology. Besides the well-described effects on the risk of hematological neoplasia, recent studies showed that CHIP mutations also accelerate the development of cardiovascular diseases such as atherosclerosis, coronary heart disease, and ischemic stroke, and worsened the outcome of heart failure patients [24]. Data from experimental studies show that mice with TET2-deficient hematopoietic cells—the first gene reported to exhibit somatic mutations in blood cells in individuals with clonal hematopoiesis without hematological malignancies [25]—display an increase in atherosclerotic plaque size [26]. These data are further supported by recent whole-exome-sequencing studies, showing that CHIP mutations are associated with an increased risk of coronary heart disease and ischemic stroke [27].

### Systemic mutations

There is evidence that patients with mutations associated with cardiomyopathies have more sensitive responses to the cardiotoxic side effects of anticancer treatments. Genome-wide association studies (GWAS) identified variants in genes, belonging to the cardiac remodeling pathway, which influence left ventricular functional changes after anthracycline exposure [28]. Most prominent are genetic variants associated with dilated cardiomyopathy, especially Titin truncating variants that increase the risk of cancer therapy-induced cardiomyopathies [29]. Therefore, genetic testing to assess the individual risk of chemotherapy cytotoxicity has been suggested [30]. Along this line, pathogenic Titin variants are frequent in patients with peripartum cardiomyopathy (PPCM; [31]) who also have a 16-fold higher risk of cancer compared with age-matched healthy women in the normal (German) population [5]. The fact that cancer occurred before as well as after the onset of PPCM underlines the complex connection between these two disease entities. Whole-exome sequencing revealed a high incidence of mutations in genes associated with either cardiomyopathies, including Titin variants, and/or the DNA damage response/repair system (DDR) in PPCM patients with a history of cancer. The prevalence of cardiomyopathy-associated gene variants was similar in PPCM patients with and without cancer but, as mentioned earlier, it may have increased the risk for subclinical cardiac damage after antitumor treatments and as a consequence the risk of developing PPCM later in life [29].

Mutations in DDR genes resulting in loss of function, downregulation, or even upregulation of the respective protein can predispose individuals to the development of cancer and other diseases [32]. The observation that DDR mutations appeared almost exclusively in PPCM patients with a history of cancer may, on one hand, explain that both disease entities emerged in these patients and, on the other hand, may point to a potential role of DDR genes in the protection of the maternal heart from pregnancy-induced stress. Furthermore, DDR mutations may also increase the risk of cardiotoxicity from antitumor therapies, as seen, e.g., in breast cancer patients with BRCA1 and BRCA2 mutations who have a higher risk of cardiac toxicity following anthracycline-based chemotherapy [33]. In addition, the relevance of DDR targets, e.g., DNA damage-inducible transcript 4 (Ddit4), for pathogenic alterations in pregnancy, for instance, of preeclampsia, a pregnancy-specific hypertensive disorder, has also been described [34]. Thus, mutations in DDR genes, which predispose to cancer, might also become relevant in the heart during phases of increased cardiac stress such as pregnancy or delivery and may thereby contribute to the development of preeclampsia and PPCM. Based on the potential influence of gene variants associated with cardiomyopathies and/or CPS, patient derived iPSC models [39] should be further explored for pretherapeutic pharmacogenetic testing [40] and personalized treatment concepts, to enhance efficacy of anti-tumor treatment and minimize adverse side effects on the cardiovascular system.

## Multi-hit hypothesis

**Fig. 1:**
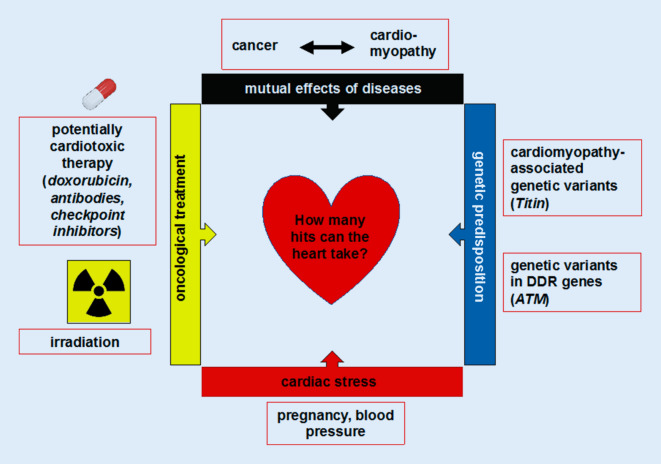
Multi-hit hypothesis in oncocardiology. Combination of multiple “hits” from oncological treatment, genetic predisposition, cardiac stress events, or mutual predisposing effects of disease entities might trigger late-onset cardiotoxicity

Hence, an important aspect in the pathogenesis of cancer and cardiovascular diseases is the multi-hit hypothesis, which states that the risk of cancer and/or cardiovascular diseases increases the more of the aforementioned factors are combined in a patient. The multi-hit hypothesis is originally a well-established model for the development of cancer, proposed by Alfred Knudson [35]. In an observational study of 48 cases of retinoblastoma, he concluded that the development of retinoblastoma is caused by two mutational events [35]. Today this hypothesis is broadly accepted, elucidating the development of many forms of cancer [36, 37]. Besides explaining the pathogenesis of cancer, this hypothesis was also transferred to the field of oncocardiology (Fig. 1). The hypothesis supports the observation that cancer patients often do not show any acute cardiovascular effects, but years later cardiovascular pathologies emerge that are likely to be late effects of antitumor therapies. For example, hematologic cancer and corresponding anticancer treatment in children and adolescents has a good survival rate and persisting heart failure is not frequent. However, if a second stressor—i.e., pregnancy, diabetes, or high blood pressure—appears later in life, it may trigger late-onset cardiomyopathy in these patients [38].

## Conclusion

Genetic background plays an important role in disease predisposition for cancer and heart failure as well as for (cardio)-toxic response to antitumor therapy. In this regard, somatic (clonal hematopoiesis of indeterminate potential [CHIP]) and germline mutations (DNA damage response/repair [DDR]) associated with a higher cancer risk may also promote cardiovascular disease, on the one hand, and increase the risk for cardiotoxic effects of antitumor treatments, on the other hand. Conversely, pathophysiological alterations during genetic cardiomyopathies, i.e., metabolic impairment and altered circulation factors, may promote the development and progression of cancer and may increase the toxic effect of antitumor therapies on the heart. Therefore, genetic analyses and pretherapeutic pharmacogenetic testing, may provide important information for optimal anti-tumor therapy with minimal (cardiovascular) side effects to ensure best possible outcome for tumor patients.
